# Jumping Performance Development in Junior Single Figure Skating at International Championships and Competitions and Its Implications for Higher Risk of Acute and Overuse Injuries: A Retrospective Observational Study from 2005 to 2020

**DOI:** 10.3390/jfmk10030251

**Published:** 2025-07-01

**Authors:** Zoé Stehlin, Felix Karl-Ludwig Klingebiel, Hans-Christoph Pape, Bergita Ganse, Thomas Rauer

**Affiliations:** 1 Department of Trauma Surgery, University Hospital Zurich, 8091 Zurich, Switzerlandhans-christoph.pape@usz.ch (H.-C.P.); 2 Faculty of Medicine, University of Zurich, 8006 Zurich, Switzerland; 3 Harald Tscherne Laboratory for Orthopaedic and Trauma Research, Department of Surgical Research, Zurich University Hospital, 8091 Zurich, Switzerland; 4 Department of Trauma, Hand and Reconstructive Surgery, Saarland University, 66421 Homburg, Germany; bergita.ganse@uks.eu; 5 Innovative Implant Development (Fracture Healing), Departments and institutes of Surgery, Saarland University, 66421 Homburg, Germany

**Keywords:** performance, figure skating, winter sports, junior athlete, trauma, age, competition

## Abstract

**Background**: Although the difficulty level of figure skating programs has increased in the last two decades, particularly at the junior level, trends in performance have not been reported. This retrospective observational study investigated performance development trends among the top five junior figure skaters competing at international levels in both the ladies’ and men’s singles disciplines from 2005 to 2020. Data from 160 junior single ladies and 160 junior single men were analyzed. The focus was on the progression of technical elements—particularly jumps—and their potential correlation with injury risk. It was hypothesized that younger athletes are increasingly performing jumps with more revolutions, thereby enhancing overall competition standards. **Materials and Methods**: Using data from the Junior World Championships and Junior Grand Prix Finals, linear regression analysis and one-way ANOVA were conducted to track the frequency of double, triple, and quadruple jumps, as well as trends in age development among athletes in the singles categories from 2005 to 2020. **Results**: The results indicate a significant increase in the execution of higher-revolution jumps among junior athletes. Between 2005 and 2012, the frequency of double jumps declined across all events, with the most pronounced reductions observed in the Ladies’ Junior World Championships (Δ = 0.216, *p* = 0.004, d = 1.64) and the Men’s Junior World Championships (Δ = 0.500, *p* = 0.001, d = 1.82). From 2005 to 2011, the frequencies of triple and quadruple jumps increased, while double jumps remained stable or showed only slight increases. Triple jumps showed slight downward trends (e.g., R^2^ = 0.0202 at the Men’s Junior World Championships). Although still rare, the frequency of quadruple jumps has shown a consistent upward trend across multiple competitions. Between 2000 and 2009, all four events exhibited declining age trends, with decreases ranging from −0.029 to −0.078 years of age per year. In the subsequent decade (2010–2020), when averaged across all events, the observed difference slope (Δ = 0.014) indicated a continued decline in athlete age. **Conclusions**: In summary, increases in more difficult jumps were found, with simultaneous decreases in less difficult jumps. As jump complexity rises, a parallel increase in sport-specific injury incidence can be anticipated, highlighting the need for proactive strategies for injury prevention and athlete well-being.

## 1. Introduction

Figure skating is a prominent winter sport and a core discipline of the Olympic Winter Games, and it has long been celebrated for its beauty and athleticism [1]. However, beneath the artistry lies a physically demanding sport that exposes athletes to a high prevalence of sport-specific injuries, ranging from acute trauma to overuse conditions [2,3,4,5]. The anatomical locations most frequently injured are the ankle, knee, tibia, and hip/groin [6,7]. Overuse injuries, such as tendinitis, patellofemoral syndrome, and lower back pain, are more common in the singles disciplines, while partner disciplines such as pairs skating, ice dancing, and synchronized skating are associated with a higher incidence of acute injuries due to the nature of throws and lifts [5,8,9,10]. The career prevalence of stress fractures, a specific type of overuse injury, is notably high in figure skaters, with rates of 24% across all athletes and 33% among those training 12 or more times per week [7].

The physical demands of figure skating are particularly evident during the execution of multi-revolution jumps, where the magnitude and intensity of impact forces increase with the number of revolutions [5,9]. In multi-revolution figure skating jumps, the time available for force dissipation upon landing decreases as the number of rotations increases, even though jump height and vertical take-off velocity remain relatively consistent across single, double, and triple jumps. Jumps with more rotations, such as triple and quadruple jumps, tend to bring skaters closer to the ice at landing due to prolonged rotational duration. This often leads to a more abrupt, collision-like impact. As a result, the landing forces are typically greater in magnitude and intensity, and if not dissipated rapidly, may substantially elevate the risk of injury [11,12]. As figure skating programs continue to grow in difficulty, the number of revolutions in jumps has also increased at junior international competitions. These evolving performance trends provide an opportunity to study how increasing jump complexity impacts both athletic performance and injury risk. In this context, jump performance (specifically regarding the number of rotations performed) offers a reliable metric for assessing progression in the sport, as previously described by our group, given the lack of time- or distance-based performance indicators in figure skating [5,9].

The current literature offers some valuable insights into injury patterns and possible mechanisms, as well as considerations for injury prevention, in the ever-evolving sport of figure skating [13]. However, little research has been conducted on performance trends in figure skating, particularly with regard to their implications for injury risk [5,9,10,13]. For example, there are hardly any studies focusing on performance development trends in junior single figure skaters at international championships. Understanding these trends is critical for assessing how changes in performance levels correspond to shifts in injury profiles—particularly given the trend toward earlier specialization in the sport and the increased physical demands on young athletes as performance volumes rise, which may increase the risk of sports and overuse injuries [14,15,16,17,18]. The objective of this study is to address the paucity of research on this sport.

In this study, we analyzed performance development trends in junior single figure skaters at international championships, focusing on the evolution of jump difficulty and the corresponding risks of both acute and overuse injuries. Using data from the Junior World Championships and Junior Grand Prix Finals, we hypothesized that performance levels have increased over time, with a concurrent decrease in the average age of top-performing athletes. This analysis aims to contribute to a deeper understanding of how the growing difficulty of jumps and the younger age of elite competitors may influence the risk of injury, to further fill the gap in the available literature on performance development trends in junior single figure skaters. It also provides insights into the management of athlete health and performance in this demanding sport.

## 2. Material and Methods

Ethical approval was obtained from the Institutional Review Board (IRB) of the Saarland Medical Board (Ärztekammer des Saarlandes; application number 135/21). The requirement for informed consent was waived by the relevant ethics committee.

### 2.1. Regulations and Judging System

The regulations and judging system used in figure skating, particularly at the World and European Championships, are governed by the International Skating Union (ISU) [19]. In the 2004/2005 season, the ISU introduced a new judging system, replacing the long-standing 6.0 scoring system that had been used since the 1891 European Championships in Hamburg. To ensure comparability, this analysis focuses on results post-2005, when the new system emphasized jumps with more rotations, altering competitive strategies. The ISU Judging System combines a base value with the Grade of Execution (GOE) to compute the Technical Elements Score (TES) [20]. While the GOE remains subjective and potentially variable across judges, this study focuses on the objective measurement of jump rotations. These are assessed by a technical panel, which includes two technical specialists and a technical controller, ensuring an impartial evaluation of jump execution based on a rigorous set of technical rules. The terminology used in the regulations refers to ‘Ladies’ (L) for female athletes and ‘Men’ (M) for male athletes. This objective approach to jump evaluation reduces subjectivity and enhances the consistency of technical assessments in modern figure skating competitions.

### 2.2. Figure Skating Disciplines and Their Specifications

At the international level, figure skating consists of five disciplines: ladies’ singles, men’s singles, pair skating, ice dancing, and synchronized skating. Each discipline requires a diverse range of skills, including multi-revolution jumps in the singles events, acrobatic lifts and throws in pair skating, aesthetic and athletic dance routines in ice dance, and coordinated group performances involving up to 20 skaters in synchronized skating. This study focused specifically on the singles disciplines, as they involve a greater variety of jumps compared to other figure skating disciplines, which are limited by scoring and performance evaluation criteria. Additionally, the kinematics of throw jumps in pair skating differ significantly from those of the jumps performed in singles disciplines, further justifying the focus of this analysis on the individual performance aspects of ladies’ and men’s singles.

### 2.3. Jumps and Revolutions

Jumps are one of the defining features of the singles disciplines in figure skating, with their difficulty increasing as the number of rotations in the air rises. This increased difficulty makes the number of rotations a key indicator of not only performance level, but also of the physical demands exerted on the athlete’s body. In singles figure skating, there are six main types of jumps: Toe Loop, Loop, Salchow, Flip, Lutz, and Axel, each of which can be executed with two, three, or four rotations. During competitions, athletes must perform a total of seven jumps, including no more than two identical jumps. The structure of jump sequences and combinations is governed by the International Skating Union (ISU) Special Regulations and Technical Rules, which determine the scoring value of each element and how different combinations affect the overall score. The current rules also specify the required number of jumping elements in a well-balanced Free Skating program. While the specific type and number of jumps—whether double, triple, or quadruple—are determined by the athletes’ ability and the choreography of their program, athletes strategically balance the potential benefits of more difficult jumps against the physical risks, taking into account their personal strengths and preferences [5]. During competitions, the jumps are assessed by a technical panel to ensure an impartial evaluation of jump execution based on a rigorous set of technical rules. In this study, falls and under-rotated or downgraded jumps were not considered. 

### 2.4. Data Acquisition

This retrospective, observational study analyzed the frequency of double, triple, and quadruple jumps performed by the top five athletes in both the ladies’ and men’s singles disciplines in the free skating programs, as well as the athletes’ ages, using data obtained from the official results databases of the Junior World Championships and Junior Grand Prix Finals from 2005 to 2020. The selection of these two competitions was made to enhance the generalizability of the findings across major international junior-level events. The analysis was constrained to the top five athletes in each competition and category, as performance differences between top-ranked and lower-ranked skaters are substantial. This enabled the analysis of the results of datasets including 160 junior single ladies and 160 junior single men. The datasets analyzed during this study comprise third-party data, which are publicly available and can be found on the website of the International Skating Union (ISU): https://www.isu.org/events, accessed on 29 April 2025. Others can access these data in the same manner as the authors, who did not have any special access privileges. The data were manually extracted by one author and checked by a second author to control for transmission errors. 

Only the definitive jump performance recognized by the technical jury was considered and evaluated as established in a previous study [5].

### 2.5. Statistical Analyses

Throughout the period of data assessment and analysis, a series of group meetings were convened to evaluate the plausibility of the data.

Analyses were conducted in R (https://www.r-project.org/) using base linear modeling functions. Figures were created in MS Excel. Significance was assumed at *p* < 0.05. Normal distributions of the data were assessed visually using histograms and Q-Q plots; no formal statistical tests for normality were applied. Effect size was calculated using Cohen’s d to estimate the magnitude of changes in average age and jump frequency between two time periods.

Linear regression was used to estimate the annual change (slope) in age and jump frequency for each jump type (double, triple, quadruple) across four junior international figure skating competitions in the singles disciplines. Separate models were fitted for the periods of 2005–2011 and 2012–2020 to assess changes in performance trends over time. For each period, the average slope was computed across all jump types and competitions. The difference between these mean slopes (Δ) was used to quantify the shift in overall jump progression trends between decades.

## 3. Results

For both the Junior World Championships and the Junior Grand Prix Finals, datasets comprising 80 athletes each in the ladies’ and men’s singles categories were analyzed. Between 2000 and 2009, the mean age of competitors in the junior ladies’ singles category at the Junior World Championships and Junior Grand Prix Finals was 15.4 years, whereas the corresponding mean age of competitors in the junior men’s singles category was 17.3 years. In the subsequent decade, from 2010 to 2020, the average age decreased to 14.4 years for junior ladies and to 16.5 years for junior men at the same international events.

### 3.1. Age Trends

In the period between 2000 and 2009, the mean age of junior athletes at the highest level of competition showed a moderate downward trend. The rate of decline ranged from –0.029 to –0.078 years per year. In the subsequent decade (2010–2020), this decline continued, albeit at varying rates depending on the event. The L Junior World Championships exhibited a marginal flattening of the age decline (Δ = –0.019, *p* = 0.02, d = 1.00), while the M Junior World Championships demonstrated a more precipitous decrease (Δ = 0.070, *p* = 0.033, d = 0.89). The L and M Junior Grand Prix Finals exhibited almost identical alterations in slope (Δ = 0.022), with substantial statistical substantiation (*p* = 0.000 and *p* = 0.014, respectively) and remarkably substantial effect sizes. When averaged across all events, the slope difference (Δ = 0.014) suggested that athletes’ ages continued to decrease at the highest levels, but the overall pace of age decline has slightly increased over the past two decades (Table 1).

**Figure 1:**
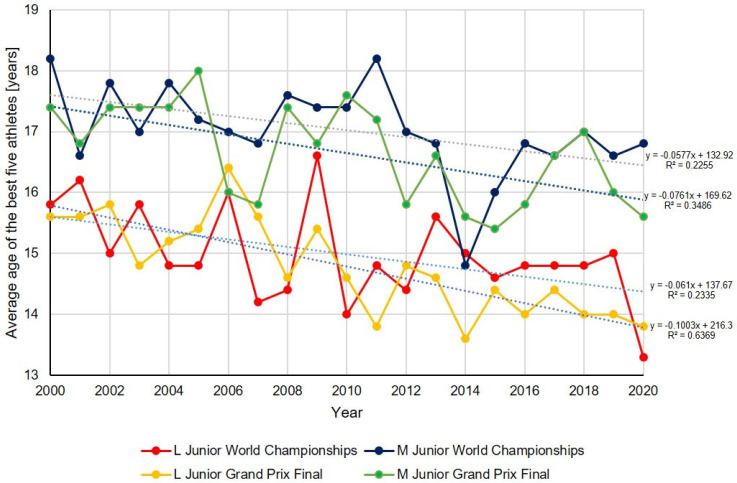
The age trends of the five best athletes in the analyzed competitions.

The average age of the top five junior figure skaters underwent a steady decline across all major international events between 2000 and 2020. While the trend was visible across various competitions, the most precipitous decline was observed at the L Junior Grand Prix Final, where the average age exhibited the most marked decrease over time (slope: –0.100 years per year, R^2^ = 0.6369). In the other events, the decreases were more moderate but consistent, with similar negative slopes for the L Junior World Championships, M Junior World Championships, and M Junior Grand Prix Final. While the overall trend indicated that younger athletes were increasingly achieving top positions, the variability between years remained relatively high, particularly in the male categories. Collectively, the data demonstrate a discernible generational transition toward younger athletes attaining peak performance in junior figure skating over the past two decades (Figure 1).

### 3.2. Numbers of Jumps

An analysis of jump performance trends from 2005 to 2020 revealed improvements across both men’s and women’s junior competitions. A decline in the frequency of double jumps was evident in all events, with the most significant decreases observed at the L Junior World Championships (Δ = 0.216, *p* = 0.004, d = 1.64) and the M Junior World Championships (Δ = 0.500, *p* = 0.001, d = 1.82). Conversely, triple jumps exhibited a marked plateauing or slight regression, particularly in the female events, where substantial negative effect sizes were evident despite negligible changes in slope. Quadruple jumps, which had been largely absent prior to 2012, began to appear with greater frequency after that year, although the overall impact on performance trends remained limited. The results of this study indicate a transition towards more technically ambitious programs, but also suggest a slowing or reversal in the traditional pathway of technical progression, especially in women’s events (Table 2).

**Figure 2:**
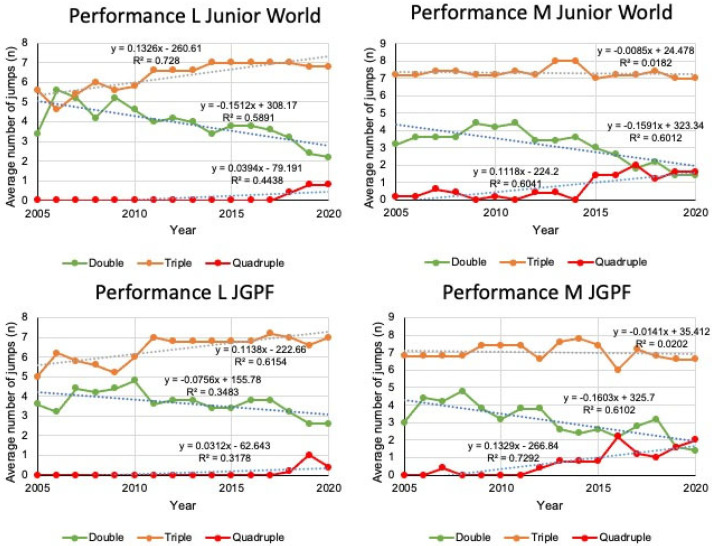
The performance trends from 2005 to 2020 in different competitions.

Between 2005 and 2020, there was a marked increase in the prevalence of specific technical shifts in the jump performance profiles of top junior athletes, as evidenced by significant linear trends across multiple events. Double jumps consistently declined over time, particularly in the men’s Junior Grand Prix Final, where the regression slope was steep (slope = –0.1603, R^2^ = 0.6102), indicating a substantial drop in double jump usage. Triple jumps remained relatively stable across events, exhibiting only minor downward trends (e.g., R^2^ = 0.0202 in the men’s Junior World Championships), suggesting that they have become the technical standard. Although still infrequent, quadruple jumps showed a consistent increase in frequency across various competitions, particularly in the men’s Junior World and Grand Prix Finals, as indicated by positive slopes and moderate R^2^ values (up to 0.6041). Compared to female skaters, male skaters demonstrated a more precipitous decline in double jumps and a more marked increase in quadruple jump attempts, suggesting a more rapid and assertive shift toward advanced technical difficulty. Overall, the statistical analysis revealed a gradual transition toward more complex jump repertoires in junior skating, with the degree of explained variance ranging from moderate to high depending on the event and jump type (Figure 2).

## 4. Discussion

The current literature suggests that early intensive training and sport specialization prior to puberty are not essential for achieving elite athletic performance in most sports [17,18,21]. Moreover, early specialization has been associated with an increased risk of overuse injuries, heightened psychological stress, and a greater likelihood of early sport discontinuation [17,18,21]. Although specializing in a sport before the end of adolescence is not recommended from a sports medicine perspective, early specialization is becoming increasingly common in modern youth sports, especially in individual sports with high coordination requirements (figure skating, ballet, rhythmic gymnastics, artistic gymnastics, and so on) [16,22,23]. It is imperative to comprehend the relationship between increased performance volume and elevated physical demands in young athletes, as this may potentially augment the risk of sports injuries and overuse injuries. Consequently, analyzing these trends is crucial to assess how changes in performance levels are associated with shifts in injury profiles [14,15,16,17,18].

In this respect, this study yielded the following main results:

- Junior athletes are demonstrating enhanced performance at a younger age;
- Technical demands have increased despite decreases in basic jump frequencies.

Sex-specific trends in figure skating show stagnation in ladies’ triple jump progression, while men’s quadruple jumps have increased significantly, indicating divergent developmental trajectories. The current literature suggests that early specialization in a sport is associated with higher training volumes and repetitive, sport-specific movement patterns, which can place considerable physical strain on young athletes if age-appropriate recovery phases are not observed [16,18,24]. This contributes to the heightened risk of sports injuries and overuse injuries [14,15,16,18,22,25,26]. In order to develop effective primary prevention programs, it is first necessary to identify relevant risk factors for the respective sport [27]. This requires considering the entire breadth of the load spectrum, including trends in performance development and the age profile of the athletes [28,29].

This study observed a decline in the average age of top-performing athletes across all junior-level competitions from 2000 to 2020. Simultaneously, the complexity of the jumps increased between 2005 and 2020, while the average number of jumps plateaued due to restrictions imposed by the International Skating Union (ISU) regulations. Evidence suggests an acceleration in the process of specialization and the attainment of enhanced performance at younger ages. Jumps with a higher number of rotations are associated with increased impact forces and greater landing intensity [11,12]. In the field of figure skating, the combination of escalating technical complexity and extended training periods has been linked to a heightened prevalence of overuse injuries and an increased risk of stress fractures [7,8]. The results of the present study indicate a faster and more assertive transition to higher levels of technical complexity in junior figure skating. By contrast, performance levels in the disciplines of sprint and long-distance running have exhibited only a marginal increase [30], while in other sports, performance has remained static or even declined [31,32,33,34,35].

Furthermore, the present study demonstrated, in the context of sex-specific trends, a stagnation or even reversal in the development of the triple jump in ladies’ figure skating events. Conversely, there has been a more significant increase in the adoption of the quadruple jump in men’s events. These differences underscore the need for sex-specific athlete monitoring for injury prevention [36,37].

The present study is the first to examine the current trends in performance and age development in international figure skating within the singles categories at the junior level.

### 4.1. Implications for Injuries and Overuse Symptoms

In figure skating, early sport specialization—combined with increasing technical jump complexity, sex-specific differences, and prolonged training durations—has been associated with a higher prevalence of both acute and overuse injuries, as jumps with more rotations are known to generate greater and more intense impact forces [7,8,11,12,38]. A higher incidence of overuse injuries has been observed in singles skating, while acute injuries are more prevalent in pairs, ice dancing, and synchronized skating [8]. Across all disciplines, lower extremity injuries are the most prevalent, with pairs accounting for the majority of upper extremity cases [8]. Ankle sprains are the most prevalent injury overall; patellar tendinitis is the leading overuse injury, and stress fractures are most common among female singles skaters [7,8].

Therefore, younger skaters in particular must develop specific physical attributes, such as agility, strength, and power, to meet rising technical demands while minimizing the risk of injury [12]. This approach depends on the observation of age-appropriate recovery phases, which are pivotal in mitigating such risks [16,17,18,24]. It may be incumbent upon coaches and federations to reassess the training loads, recovery strategies, and psychological support provisions for young athletes exposed to elevated levels of competitive pressure.

### 4.2. Limitations

This retrospective, observational study, despite being the first to address this topic, covers a relatively short period and a low number of athletes. Furthermore, the results may have been affected by inconsistencies arising from the judges evaluating the skaters differently. Future studies should analyze a larger cohort. 

A major limitation of this study is the lack of direct measurement of injury incidence among participants. Nevertheless, it was hypothesized that a higher frequency of jump revolutions is associated with an increased injury risk in junior single figure skaters. This assumption is supported by prior research demonstrating a temporal increase in injury rates within this population.

As the dataset analyzed in this study was retrospective–observational, the performance trajectories of individual athletes were not taken into account; however, this is a topic of interest that should be addressed in the future. In addition, the authors did not have access to data on athletes’ body size and weight; therefore, they could not analyze their effects on performance. All of these factors may affect talent identification and selection.

## 5. Conclusions

This study is the first to analyze the current trends in performance and age development among junior-level international figure skaters in the singles disciplines. The findings indicate a rise in jump complexity accompanied by a decreasing average athlete age, suggesting a shift toward earlier sport-specific specialization. The existing literature further indicates that multi-revolution jumps generate higher and more intense landing forces, which, if not dissipated efficiently, may increase the risk of injury. These trends underscore the need for closer monitoring of potential rises in acute and overuse injuries, particularly in light of evolving competition regulations. Accordingly, adjustments to training and competition loads should be considered to better support athlete health and development. Prevention programs for competitive figure skaters should be designed to align with each individual’s age and stage of physical development. These programs should emphasize the progressive refinement of technical skills alongside the targeted development of strength, conditioning, and coordination capacities to meet the escalating neuromotor, balance, and strength demands inherent in the sport.

## Figures and Tables

**Table 1:** The changes in average athlete age between the periods 2000–2009 and 2010–2020 across international junior figure skating competitions. Δ indicates the difference in the regression slope. The effect size was calculated using Cohen’s d. * indicates statistically significant differences (*p* < 0.05).

Event	Slope 2000–2009	Slope 2010–2020	Δ (Slope Difference)	*p*-Value	Effect Size (Cohen’s d)
L Junior World Championships	−0.053	−0.035	−0.019	0.020 *	1
M Junior World Championships	−0.035	−0.105	0.070	0.033 *	0.890
L Junior Grand Prix Final	−0.029	−0.051	0.022	0 *	2.791
M Junior Grand Prix Final	−0.078	−0.100	0.022	0.014 *	1.040
Average	−0.049	−0.063	0.014		

**Table 2:** The changes in jump performance (double, triple, quadruple) between the periods 2005–2011 and 2012–2020. Slopes were derived via linear regression. Δ indicates the slope difference. The effect sizes (Cohen’s d) quantify the magnitude of change. * indicates statistically significant differences (*p* < 0.05).

Event	Jump	Slope 2005–2011	Slope 2012–2020	Δ (Slope Difference)	*p*-Value	Effect Size (Cohen’s d)
L Junior World	Double	−0.007	−0.223	0.216	0.004 *	1.637
	Triple	0.2	0.023	0.177	0.999	−2.887
	Quadruple	0	0.127	−0.127	0.978	−1.059
M Junior World	Double	0.2	−0.300	0.500	0.001 *	1.819
	Triple	0.014	−0.080	0.094	0.568	−0.079
	Quadruple	−0.043	0.190	−0.233	0.998	−1.659
L Junior Grand Prix Final	Double	0.114	−0.140	0.254	0.017 *	1.231
	Triple	0.179	0.017	0.162	0.997	−2.275
	Quadruple	0	0.083	−0.083	0.923	−0.695
M Junior Grand Prix Final	Double	−0.014	−0.247	0.232	0.003 *	1.582
	Triple	0.129	−0.087	0.215	0.563	−0.078
	Quadruple	−0.014	0.160	−0.174	1	−2.430
Average		0.063	−0.04	0.103		

## Data Availability

The datasets analyzed in this study comprise third-party data, which can be found on the website of the International Skating Union (ISU): https://www.isu.org/events (accessed on 1 May 2024).

## References

[1] Soligard T., Steffen K., Palmer-Green D., Aubry M., Grant M.E., Meeuwisse W., Mountjoy M., Budgett R., Engebretsen L. (2015). Sports Injuries and Illnesses in the Sochi 2014 Olympic Winter Games. Br. J. Sports Med..

[2] Fortin J.D., Roberts D. (2003). Competitive Figure Skating Injuries. Pain. Physician.

[3] Porter E.B. (2013). Common Injuries and Medical Problems in Singles Figure Skaters. Curr. Sports Med. Rep..

[4] Ridge S., Bruening D., Charles S., Stahl C., Smith D., Reynolds R., Adamo B., Harper B., Adair C., Manwaring P. (2020). Icesense Proof of Concept: Calibrating an Instrumented Figure Skating Blade to Measure on-Ice Forces. Sensors.

[5] Rauer T., Pape H.C., Knobe M., Pohlemann T., Ganse B. (2022). Figure Skating: Increasing Numbers of Revolutions in Jumps at the European and World Championships. PLoS ONE.

[6] Jederström M., Agnafors S., Ekegren C., Fagher K., Gauffin H., Korhonen L., Park J., Spreco A., Timpka T. (2021). Determinants of Sports Injury in Young Female Swedish Competitive Figure Skaters. Front. Sports Act. Living.

[7] Andrew Naylor T., Naylor S. (2023). Distribution and Risk Factors for Stress Fractures in Competitive Figure Skaters and Association with Acute Fractures. Phys. Sportsmed..

[8] Han J.S., Geminiani E.T., Micheli L.J. (2018). Epidemiology of Figure Skating Injuries: A Review of the Literature. Sports Health.

[9] Rauer T., Pape H.C., Stehlin Z., Heining S., Knobe M., Pohlemann T., Ganse B. (2022). Performance Increases in Pair Skating and Ice Dance at International Championships and Olympic Games. Int. J. Environ. Res. Public Health.

[10] Dubravcic-Simunjak S., Pecina M., Kuipers H., Moran J., Haspl M. (2003). The Incidence of Injuries in Elite Junior Figure Skaters. Am. J. Sports Med..

[11] Lockwood K., Gervais P. (1997). Impact Forces Upon Landing Single, Double, and Triple Revolution Jumps in Figure Skaters. Clin. Biomech..

[12] King D.L. (2005). Performing Triple and Quadruple Figure Skating Jumps: Implications for Training. Can. J. Appl. Physiol..

[13] Dubravcic-Simunjak S., Kuipers H., Moran J., Simunjak B., Pecina M. (2006). Injuries in Synchronized Skating. Int. J. Sports Med..

[14] Post E.G., Bell D.R., Trigsted S.M., Pfaller A.Y., Hetzel S.J., Brooks M.A., McGuine T.A. (2017). Association of Competition Volume, Club Sports, and Sport Specialization with Sex and Lower Extremity Injury History in High School Athletes. Sports Health.

[15] Post E.G., Trigsted S.M., Riekena J.W., Hetzel S., McGuine T.A., Brooks M.A., Bell D.R. (2017). The Association of Sport Specialization and Training Volume with Injury History in Youth Athletes. Am. J. Sports Med..

[16] DiFiori J.P., Benjamin H.J., Brenner J., Gregory A., Jayanthi N., Landry G.L., Luke A. (2014). Overuse Injuries and Burnout in Youth Sports: A Position Statement from the American Medical Society for Sports Medicine. Br. J. Sports Med..

[17] Jayanthi N.A., LaBella C.R., Fischer D., Pasulka J., Dugas L.R. (2015). Sports-Specialized Intensive Training and the Risk of Injury in Young Athletes: A Clinical Case-Control Study. Am. J. Sports Med..

[18] Jayanthi N.A., Post E.G., Laury T.C., Fabricant P.D. (2019). Health Consequences of Youth Sport Specialization. J. Athl. Train..

[19] International Skating Union Website of the International Skating Union (Isu). https://www.isu.org.

[20] Hirosawa S., Watanabe M., Aoki Y. (2022). Determinant Analysis and Developing Evaluation Indicators of Grade of Execution Score of Double Axel Jump in Figure Skating. J. Sports Sci..

[21] Jayanthi N., Pinkham C., Dugas L., Patrick B., LaBella C. (2013). Sports Specialization in Young Athletes:Evidence-Based Recommendations. Sports Health.

[22] Pasulka J., Jayanthi N., McCann A., Dugas L.R., LaBella C. (2017). Specialization Patterns across Various Youth Sports and Relationship to Injury Risk. Phys. Sportsmed..

[23] Law M.P., Côté J., Ericsson K.A. (2007). Characteristics of Expert Development in Rhythmic Gymnastics: A Retrospective Study. Int. J. Sport Exerc. Psychol..

[24] Lloyd R.S., Cronin J.B., Faigenbaum A.D., Haff G.G., Howard R., Kraemer W.J., Micheli L.J., Myer G.D., Oliver J.L. (2016). National Strength and Conditioning Association Position Statement on Long-Term Athletic Development. J. Strength. Cond. Res..

[25] LaPrade R.F., Agel J., Baker J., Brenner J.S., Cordasco F.A., Côté J., Engebretsen L., Feeley B.T., Gould D., Hainline B. (2016). Aossm Early Sport Specialization Consensus Statement. Orthop. J. Sports Med..

[26] Brenner J.S. (2016). Sports Specialization and Intensive Training in Young Athletes. Pediatrics.

[27] van Mechelen W. (1997). Sports Injury Surveillance Systems. ‘One Size Fits All’?. Sports Med..

[28] Emery C.A., Pasanen K. (2019). Current Trends in Sport Injury Prevention. Best. Pract. Res. Clin. Rheumatol..

[29] Steffen K., Emery C.A., Romiti M., Kang J., Bizzini M., Dvorak J., Finch C.F., Meeuwisse W.H. (2013). High Adherence to a Neuromuscular Injury Prevention Programme (Fifa 11+) Improves Functional Balance and Reduces Injury Risk in Canadian Youth Female Football Players: A Cluster Randomised Trial. Br. J. Sports Med..

[30] Weiss M., Newman A., Whitmore C., Weiss S. (2016). One Hundred and Fifty Years of Sprint and Distance Running—Past Trends and Future Prospects. Eur. J. Sport. Sci..

[31] Berthelot G., Tafflet M., El Helou N., Len S., Escolano S., Guillaume M., Nassif H., Tolaïni J., Thibault V., Desgorces F.D. (2010). Athlete Atypicity on the Edge of Human Achievement: Performances Stagnate after the Last Peak, in 1988. PLoS ONE.

[32] Ganse B., Degens H. (2021). Declining Track and Field Performance Trends in Recent Years in the Austrian Best Results 1897–2019. J. Musculoskelet. Neuronal Interact..

[33] Lippi G., Banfi G., Favaloro E.J., Rittweger J., Maffulli N. (2008). Updates on Improvement of Human Athletic Performance: Focus on World Records in Athletics. Br. Med. Bull..

[34] Marck A., Antero J., Berthelot G., Saulière G., Jancovici J.M., Masson-Delmotte V., Boeuf G., Spedding M., Le Bourg É., Toussaint J.F. (2017). Are We Reaching the Limits of Homo Sapiens?. Front. Physiol..

[35] Nevill A.M., Whyte G. (2005). Are There Limits to Running World Records?. Med. Sci. Sports Exerc..

[36] Tomkinson G.R., Carver K.D., Atkinson F., Daniell N.D., Lewis L.K., Fitzgerald J.S., Lang J.J., Ortega F.B. (2018). European Normative Values for Physical Fitness in Children and Adolescents Aged 9–17 Years: Results from 2,779,165 Eurofit Performances Representing 30 Countries. Br. J. Sports Med..

[37] Beynnon B.D., Tourville T.W., Hollenbach H.C., Shultz S., Vacek P. (2023). Intrinsic Risk Factors for First-Time Noncontact Acl Injury: A Prospective Study of College and High School Athletes. Sports Health.

[38] King D., Smith S., Higginson B., Muncasy B., Scheirman G. (2004). Characteristics of Triple and Quadruple Toe-Loops Performed During the Salt Lake City 2002 Winter Olympics. Sports Biomech..

